# The Protective Role of Cognitive Reserve in Mild Cognitive Impairment: A Systematic Review

**DOI:** 10.3390/jcm12051759

**Published:** 2023-02-22

**Authors:** Ilaria Corbo, Giulia Marselli, Valerio Di Ciero, Maria Casagrande

**Affiliations:** 1 Dipartimento di Psicologia, Università di Roma Sapienza, 00185 Roma, Italy; 2 Dipartimento di Psicologia Dinamica, Clinica e Salute, Università di Roma Sapienza, 00185 Roma, Italy

**Keywords:** cognitive reserve, mild cognitive impairment, aging

## Abstract

Cognitive reserve (CR) represents the ability to optimize performance and functioning to cope with brain damage or disease. CR reflects the capability to adaptively and flexibly use cognitive processes and brain networks to compensate for the deterioration typical of aging. Several studies have investigated the potential role of CR in aging, especially from the perspective of preventing and protecting against dementia and Mild Cognitive Impairment (MCI). This systematic literature review aimed to investigate the role of CR as a protective factor against MCI and associated cognitive decline. The review process was conducted according to the PRISMA statement. For this purpose, ten studies were analyzed. The results of this review show that high CR is significantly associated with a reduced risk of MCI. In addition, a significant positive relationship between CR and cognitive functioning is observed when comparing subjects with MCI and healthy subjects and within people with MCI. Thus, the results confirm the positive role of cognitive reserve in mitigating cognitive impairment. The evidence from this systematic review is consistent with the theoretical models of CR. Indeed, previous research hypothesized that specific individual experiences (such as leisure activities) allow a person to acquire successful neural resources over the years to cope with cognitive decline.

## 1. Introduction

Cognitive reserve represents a latent construct, initially defined as the ability of the brain to optimize and maximize performance and functioning through the recruitment of specific networks and the use of alternative cognitive strategies in order to cope with brain damage or pathology [1]. This construct was theorized to explain the observed discrepancy between the severity of brain pathologies and their clinical manifestations [2]. The term cognitive reserve was then introduced to describe individual differences, in terms of neural and cognitive resources, that allowed some individuals, compared to others, to maintain a better level of functioning in the presence of brain damage [2]. Stern and coworkers [3] have recently defined cognitive reserve as the adaptability of cognitive and neural networks to optimize individual functioning in healthy and cognitively impaired older adults [3]. Cognitive reserve would, thus, reflect the ability to adaptively and flexibly use cognitive processes and brain networks to compensate for the deterioration typical of healthy or pathological aging [3,4]. Cognitive reserve can be distinguished from brain reserve; the latter is defined as the set of structural features (such as brain volume, number of synapses, and white matter integrity) of the brain at any given time [5]. Such structural features would play a protective role in cognitive decline in healthy and pathological aging [5,6].

Since these early observations, several studies have investigated the potential role played by cognitive reserve in healthy and pathological aging, especially from a preventive and protective perspective of dementia development [5,6,7,8,9]. These studies suggest the role of cognitive reserve as a protective factor that can mitigate cognitive decline in healthy and pathological aging [2,4]. People with a high cognitive reserve are more able to tolerate the presence of brain pathology and delay or halt the onset of subsequent cognitive impairment [6]. Increasing interest has been observed over the years regarding the role played by cognitive reserve in Mild Cognitive Impairment (MCI).

MCI is a syndrome characterized by a moderate decline in cognitive functioning. This condition does not meet the diagnostic criteria for dementia but is characterized by cognitive impairment worse than expected for age [10]. MCI can be distinguished into four types based on the number (single or multiple domains) and the type of cognitive domains involved (amnesic or nonamnesic type). Amnesic MCI is defined by memory impairment that can be single-domain (aMCI) or multiple domains (aMCI-md) when cognitive deficits other than memory deficit are presented. When subjects do not have a memory deficit, we refer to nonamnesic MCI, which can be single-domain (naMCI) or multiple-domains (naMCI-md), depending on the number of cognitive functions involved [2,3]. Individuals with MCI can exhibit a range of cognitive impairments, such as memory and executive deficits [11,12,13], but they also manifest psychological and behavioral symptoms, such as sleep disorders [14]. MCI may represent the prodromal stage of dementia, but at the same time, it can be a reversible condition [15], and it is considered a “window” during which it is still possible to intervene to avoid or delay the onset of dementia [16]. In this condition, cognitive reserve could be an important element in reducing the risk of developing MCI and its subsequent conversion into dementia.

Several studies have investigated the role of cognitive reserve in MCI to understand whether it can reduce the cognitive impairment characteristic of this condition, delaying or halting its conversion into dementia. Accordingly, many studies have highlighted the role played by years of schooling, leisure time, and work activity as protective factors for cognitive decline [17,18,19,20].

In particular, Nelson et al. [9] showed that brain capacity is only partially dependent on neuropathology because higher levels of cognitive reserve (socio-behavioral factors) were significantly associated with a reduced risk of developing MCI. Berezuk et al. [21] found that high levels of cognitive reserve were associated with better cognitive functioning in individuals with MCI. This relationship was especially observed when considering certain indices of cognitive reserve, such as job position and schooling. In addition, an interesting relationship was found between cognitively and socially stimulating activities and good daily functioning in subjects with MCI. Although these meta-analyses [9,21] investigated the role of cognitive reserve in MCI, they have some limitations. Nelson et al. [9] did not consider cross-sectional studies and focused on the role of cognitive reserve in the progression from MCI to dementia. On the other hand, Berezuk et al. [21] did not consider longitudinal studies and included only studies without a healthy control group; therefore, it cannot be concluded whether people with MCI had a lower cognitive reserve than healthy elderly people.

In conclusion, the importance and usefulness of cognitive reserve in preventing cognitive impairment still need to be clarified in its definition and operationalization [5] and can be useful to delineate its role in MCI better.

This systematic review aims to summarize the current state of the literature concerning the relationship between cognitive reserve and MCI, considering the protective role of cognitive reserve in MCI. Considering the limitations observed in the two previous meta-analyses, we focused on cross-sectional and longitudinal studies, including both individuals with MCI and healthy elderly control groups. These choices allow us to delineate a more comprehensive picture of the relationship between cognitive reserve and MCI.

Based on previous studies, we expect individuals with MCI to have lower cognitive reserve than healthy older adults, confirming, albeit indirectly, the protective role of cognitive reserve in preventing or slowing cognitive impairment.

## 2. Materials and Methods

The review process was conducted according to the PRISMA Statement 2020 [22].

### 2.1. Research Strategies

A systematic search was conducted in the following databases, Psycinfo, MEDLINE, Scopus, and Web of Science, selecting articles in peer-reviewed journals. The last search was conducted on 28 December 2022.

Restrictions were made, limiting the research to academic publications with English or Italian full text, without restrictions regarding years, gender, and ethnicity. Additionally, the bibliographical references of retrieved papers, reviews, and meta-analyses were screened manually to assess whether they included relevant studies to include in the review. The scripts used and the number of selected articles are shown in Table 1.

### 2.2. Eligibility Criteria

Articles selected on the basis of the initial script were reviewed by two researchers in order to include only those studies that met the following eligibility criteria: randomized controlled, cross-sectional, and longitudinal studies that had as their primary or secondary objective to assess the level of cognitive reserve and its relationship to cognitive functioning in healthy individuals and patients with MCI; studies in which the diagnostic criteria of MCI were clearly defined and in which the diagnosis of MCI was made with standardized clinical or neuropsychological instruments; studies where the assessment of cognitive reserve and cognitive functioning have been made with standardized clinical instruments; participants with age equal or higher than 50; and studies that clearly reported how participants were recruited.

The exclusion criteria were: studies including patients with head injury, neurological diseases (e.g., multiple sclerosis, Parkinson’s disease and epilepsy), metabolic diseases such as diabetes or metabolic syndrome, autoimmune diseases, such as rheumatoid arthritis or lupus, cardiovascular diseases, such as stroke or heart attack, oncological diseases, patients diagnosed with frontotemporal dementia, vascular dementia and Lewy body dementia, patients with Alzheimer’s disease, patients diagnosed with psychiatric disorders; studies that included subjects with subjective memory disorders diagnosis, cognitive decline diagnosis based on the caregiver assessment, or cognitive decline diagnosis other than MCI (cognitive impairment non dementia-CIND, age-associated memory impairment-AAMI, age-associated cognitive decline-AACD); studies where the diagnosis of MCI was made based only on neurophysiological tools, neuroimaging, or biological markers; studies considering animal models; reviews, meta-analyses, editorials, commentaries, posters, conferences, single-cases, clinical trials.

### 2.3. Data Collection

The first search resulted in the selection of 5922 articles from the four databases used. After the elimination of duplicates (515), carried out with the Mendeley program, the title and abstract of the remaining 5407 articles were read. Over 5000 articles were excluded based on reading the title and abstract because they did not consider older adults with MCI. This exclusion led to the selection of only 69 full texts.

The reading of these 69 articles led to the exclusion of 31 articles because the control group was missing, 18 articles did not include a sample with MCI, and 10 articles did not measure CR adequately. At the end of this process, ten articles, which met the previously described inclusion criteria, were selected for this systematic review.

**Figure 1:**
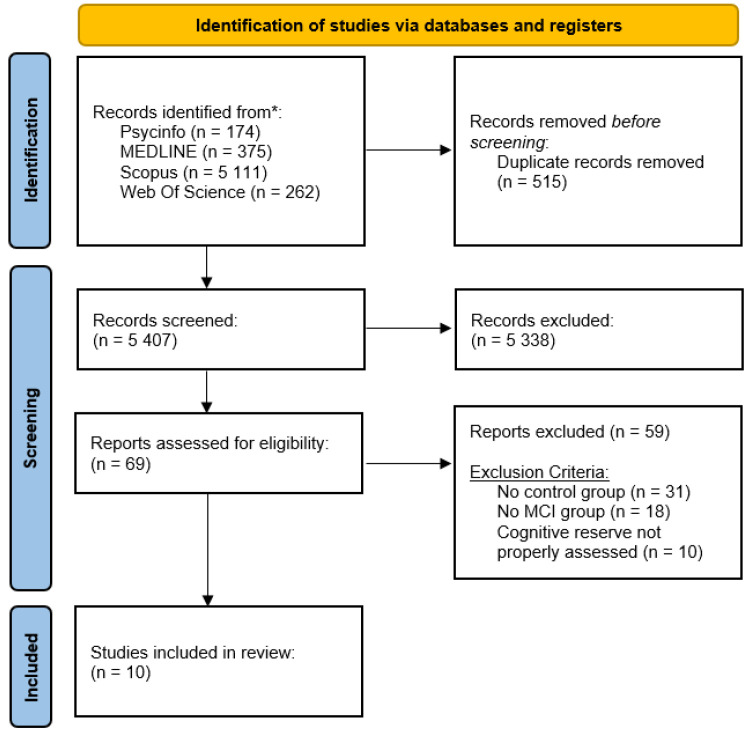
PRISMA 2020 Flow Chart.

The PRISMA 2020 flow chart [22], observable in Figure 1, summarizes this data collection.

According to the PICOS approach [23], the following information was extracted from each of the articles included in the review: authors and year of publication, nationality, experimental design, number and characteristics of participants (age, gender), the diagnostic criteria used for the diagnosis of Mild Cognitive Impairment (MCI), cognitive reserve assessment, cognitive functioning assessment, and results.

### 2.4. Quality Assessment

The Cochrane Collaboration’s tool for assessing the risk of bias was adapted to conduct a quality assessment of the selected studies [24].

For this systematic review, we considered the following risks of bias:

(I) attrition bias (i.e., biases caused by the utilization of incomplete outcome data);
(II) reporting bias (i.e., bias due to selective outcome reporting or not reporting relevant results);
(III) sample bias (i.e., bias resulting in samples that do not represent the general population, undermining the generalization of results, or articles reporting scarce demographic information, such as the male/female ratio, participants’ mean age, and schooling years);
(IV) measurement bias (i.e., bias due to using non-validated tasks to measure cognitive reserve or non-specific tasks to measure cognitive functioning).

## 3. Results

Ten studies were selected for this systematic review, including five longitudinal studies [25,26,27,28,29] and five cross-sectional studies [20,30,31,32,33].

The geographic distribution of the studies was as follows: four were conducted in Europe [30,31,32,33], three in Asia [25,26,29], and three in North America [20,27,28]

These studies included 6145 participants, among whom 2180 were participants with MCI and 3965 were healthy control participants.

The sample of MCI participants had a mean age ranging from 63 [28] to 83 years [20]. The healthy participants had a mean age ranging from 55.7 [28] to 80 years [20].

### 3.1. Publication Bias

A general overview of the possible risks of bias across all reviewed studies is presented in Table 2. Four studies [26,27,31,32] did not meet any considered bias. Attrition and reporting biases posed low risks in all the included studies. On the other hand, there was a high risk of sample bias in four studies [28,29,30,33], which was due to samples that did not represent the general population (small sample size and majority of female participants or younger participants in the sample). Measurement bias was rated as high risk in two studies [20,34] due to the use of non-validated tasks to measure cognitive reserve [20] and non-specific tests to measure cognitive functioning [34].

### 3.2. Diagnostic Criteria

Different criteria were used for diagnosing MCI. Specifically, four studies used Petersen’s criteria. Two out of four studies [30,32] relied on the 2004 criteria [10], one [26] on the 1999 criteria [35], and a final study [33] integrated Petersen’s 2001 criteria with Lopez’s criteria [36,37]. Winblad’s criteria [11] were used in two studies [27,31]. One study [25] used the “Diagnostic and Statistical Manual of Mental Disorders-IV—DSM-IV” criteria [38], one study [20] used the criteria of the Mayo Clinic Revised [39], another study [28] utilized the Albert et al. [40] criteria, and one study [29] referred to the “National Institute of Neurological and Communicative Disorders and Stroke-Alzheimer’s Disease and Related Disorders Association—NINCDS-ADRDA” criteria [41]. The diagnostic criteria are reported in Table 3 and Table 4

### 3.3. Cognitive Reserve Assessment

The selected studies assessed cognitive reserve by referring to different indices of the construct.

The most widely used index was the schooling level [25,26,28,29,30,31,32,33]. Other indices used were job position [25,26,30,33], leisure time participation in cognitively stimulating (e.g., reading a book) and socially interesting activities (e.g., going to the cinema and theater) [20,26,29,30,33], verbal IQ [32], and some tests that refer to some aspects of IQ, such as Raven’s matrices [27], the “National Adult Reading Test—NART” [27,28] and the Wechsler Adult Intelligence Scale-III—WAIS-III” vocabulary scale [28,30].

These indices were assessed both by interview or performance tests [20,25,28,29,30,31,33] and using structured self-report instruments such as the “Cognitive Reserve Index Questionnaire—CRI” [26] and the “Lifestyle Activities Questionnaire—LAQ” [27].

### 3.4. Cognitive Functioning Assessment

Cognitive functioning was assessed using general cognitive functioning tests and specific tests to evaluate single cognitive domains.

The general cognitive functioning was assessed by the “Consortium to Establish a Registry for Alzheimer’s Disease-Neuropsychological test battery—CERAD-NP” [29], the “Clinical Dementia Rating Scale—CDR” [27], the “Global Deterioration Scale—GDS” [25], the “Montreal Cognitive Assessment—MoCa” [25], the “Mini-Mental State Examination—MMSE” [25,30,33], and the “Wechsler Adult Intelligence Scale-Revised—WAIS-R” [28].

Other specific tasks have been used to assess more specific cognitive domains, such as memory [20,27,28,30,32,33], attention [27], language [20,27], psychomotor speed [27], executive functions [20,27,30], visuospatial skills [20,30], praxia [30], and constructive skills [27,30].

Table 5 reports the cognitive domains assessed in the studies.

### 3.5. Results of the Selected Studies

#### 3.5.1. Longitudinal Studies

All the studies considered the differences between individuals with MCI and healthy people in cognitive reserve, as well as the correlation between cognitive reserve and cognitive functioning. All the longitudinal studies [25,26,27,28,29] highlighted a significant association between a high cognitive reserve and better cognitive functioning and highlighted that people characterized by high cognitive reserve presented a lower risk of developing MCI. Liu et al. [25] found that differences in cognitive performance between participants with MCI and healthy participants correlated with some indicators of cognitive reserve. Specifically, a significant relationship was observed between years of schooling and scores on the MoCA, MMSE, and WMS and between MoCA scores and years of schooling. Malave’s [27] study found that cognitively stimulating activities, such as reading newspapers and weeklies, and IQ were associated with a lower incidence of MCI. Especially IQ appears to reduce the risk of developing MCI. Similarly, Soldan et al. [28], analyzing a sample of healthy participants, showed that individuals with a lower cognitive reserve had poor cognitive functioning and were significantly more at risk of developing MCI. In addition, it was observed that participants with high cognitive reserve, who later developed a condition of MCI, manifested better cognitive performance before the onset of symptoms than participants with low cognitive reserve; however, compared to the latter, they presented a more rapid cognitive decline after the onset of symptoms, according to cognitive reserve model proposed by Stern [2]. Similar results are confirmed by Xu et al. [29], who observed, from a longitudinal perspective, that a high level of cognitive reserve was significantly associated with a lower risk of developing MCI in the sample under study. Finally, Kim and coworkers [26] found that healthy participants, compared with participants diagnosed with MCI, had higher scores on the “CRI-leisure time” section of the CRIq and greater cognitive functioning. Table 6 reports the results of the selected studies.

#### 3.5.2. Cross-Sectional Studies

Five papers adopted a cross-sectional design [20,30,31,32,33]. The first study [30] observed a correlation between cognitive reserve and performance in an implicit associative memory task within the group of participants with MCI. Indeed, MCI participants with high cognitive reserve had better performance in this task than MCI participants with low cognitive reserve. Similarly, Solé-Padullès and coworkers [33] found that participants with MCI significantly presented lower cognitive reserve and worse performance in an amnestic recognition task than the control group. Another study [32] found that the level of cognitive reserve mediated the performance in some tests assessing episodic memory in a sample of aMCI participants. Specifically, aMCI participants with high cognitive reserve had better performance on episodic memory tasks, suggesting a protective role of cognitive reserve toward residual mnestic abilities in participants with memory impairment compared with participants with the same deficit but reduced CR [32].

A further study [20] investigated the possible protective role in the development of MCI played by being engaged in cognitively stimulating activities, assessed through a questionnaire, which asked how often the participant was engaged in these activities. The results showed that certain activities, such as reading books and newspapers, craft activities, using computers, engaging in cognitively stimulating games, and watching less television, were significantly associated with a lower risk of MCI. Finally, another study [31] focused on differences in cognitive performance between participants classified as single-domain aMCI and multiple-domain aMCI+ patients in relation to their cognitive reserve. Results highlighted that single-domain aMCI exhibited a better level of cognitive functioning, especially in executive functions, correlated with educational level, one of the main indices of cognitive reserve construct. Therefore, the authors suggested that cognitive reserve plays a compensatory role in this group of participants. The better cognitive performance of patients with single-domain aMCI compared to subjects with multiple-domain aMCI-md could be explained by the different levels of cognitive reserve [31]. Table 6 reports the results of selected studies.

## 4. Discussion

This systematic literature review aimed to investigate the role of cognitive reserve as a protective factor of MCI and its associated cognitive decline.

The selected studies differ in several aspects, and of particular interest for this review are the differences in diagnostic criteria, assessment of cognitive functioning, and cognitive reserve. Actually, there are no well-defined diagnostic criteria for the diagnosis of MCI, and those used vary from study to study [16,43,44]. On the other hand, concerning the assessment of cognitive functioning, there is great heterogeneity in the cognitive functions assessed and the instruments used for evaluating cognitive functioning [43]. Differences are also observed in the assessment of cognitive reserve. Several social–behavioral indices have been proposed to evaluate this construct [4,45]. Furthermore, the included studies presented a high variability in the assessment methods and cognitive reserve indices. These differences represent a limitation of this systematic review and will be discussed in more detail below.

Despite these differences, the results and conclusions of the different studies are similar, suggesting a potential protective role played by cognitive reserve for the development of MCI and the cognitive decline accompanying it. High levels of cognitive reserve are significantly associated with a reduced risk of MCI in longitudinal studies [25,27,29]. Cross-sectional studies [20,28] substantiate a positive association between cognitive reserve and cognitive functioning. The results were confirmed by comparing subjects with MCI and healthy subjects [26,30,33] and within the MCI population [31,32], emphasizing the role played by cognitive reserve in mitigating cognitive impairment, probably through the enactment of compensatory mechanisms [4,45].

Although social influences modulate cognitive reserve, we found no differences among the studies conducted on different continents, but this could be related to the small number of articles analyzed. Regardless of the participants’ nationality and the geographical location where the studies were conducted, all the studies showed differences between the two groups investigated and the same relationship between cognitive functioning and cognitive reserve.

Even if all the studies considered both females and males, they did not report gender differences in the prevalence of MCI, cognitive functioning, and cognitive reserve. Previous studies [46,47] found that men had higher premorbid intelligence than women and that this influenced the age of onset and the severity of cognitive decline; in women, on the other hand, it appears that education contributes less to the onset of cognitive impairment.

The findings of this systematic review are consistent with the theoretical models on cognitive reserve [4,45]. These models hypothesized that specific individual experiences, such as job position, education, and involvement in socially (e.g., going to the cinema) and cognitively (e.g., reading a book) stimulating activities, allow a person to acquire neural resources over the years [45]. Stimulating activities (e.g., reading a book, schooling years, etc.) would enhance neural resources, constituting the substrate of cognitive reserve, which allows the person to mitigate the cognitive decline due to healthy or pathological aging [2,45]. Specifically, it is hypothesized that cognitive reserve acts through two main mechanisms: neural capacity and efficiency [45]. Neural capacity refers to the neural resources available for cognition, while neural efficiency represents the ability to use these resources efficiently [45]. Through such mechanisms, cognitive reserve would compensate for the cognitive impairment observed in healthy and pathological aging [2]. Participants with a high cognitive reserve can tolerate greater levels of pathology than those with a low cognitive reserve, thereby slowing expected cognitive deterioration [2]. In the case of a pathological condition of aging, such as MCI, higher cognitive reserve could mitigate its risk and delay its onset. The results of this review support these models, highlighting and emphasizing the protective role of cognitive reserve for MCI.

### Limitations

The main limitation of this systematic review is the great methodological variability in the selected studies, which differ significantly in some aspects, which are the focus of this systematic review, such as the criteria used to diagnose MCI and the assessment of cognitive reserve and cognitive functioning.

The first aspect to consider is the diagnosis of MCI, which represents an evolving construct whose diagnostic criteria still need to be defined [16,48]. There are different diagnostic criteria [43], and this heterogeneity makes it difficult to compare the studies.

This strong heterogeneity was also found in the assessment of cognitive reserve, which is a latent construct, not directly observable but inferred from specific socio–behavioral indices such as education, job position, IQ, and participation in specific social and cognitive activities [45]. The studies considered in this review differ in the indices used and how they are assessed. This variability makes it difficult to compare the results emerging from the different studies when considering the relationship between MCI and cognitive reserve. Another limitation of this review is the wide heterogeneity of cognitive aspects analyzed. In fact, no study analyzed all cognitive domains, while others analyzed only one aspect of cognition [25,26,29,31,33]. Certainly, it would be interesting to observe the effects of cognitive reserve on more complex cognitive functions, such as simple and higher executive functions, which have been little or not at all considered, and which are often impaired in older people with MCI [12,13]. In many cognitive domains, such as attention, only tasks assessing simple aspects were considered (e.g., Trail Making Test-A). Concerning attention, it would be useful to evaluate different aspects of attentional networks and their interactions (e.g., [49,50]). It could also be interesting to evaluate whether the relationship between cognitive functioning and cognitive reserve is mediated by other conditions associated with aging, such as high blood pressure [51] and poor sleep quality [14]. Another limitation is the absence of statistical matching in the reviewed studies; none controlled for variations in demographic, social, or biological factors other than cognitive reserve in comparing the groups.

Finally, an additional aspect that may have limited the results was the assessment only of individuals with MCI, excluding subjects with dementia. This aspect needs to be investigated better in the future to observe how CR impacts the entire continuum from healthy to severely pathological aging.

## 5. Conclusions

Considering the general population’s aging, increased dementia, and the absence of effective pharmacological treatments [52], it is crucial to act on the prevention front. MCI represents a transitional phase from cognitive aging to dementia and is considered a stage during which it is still possible to intervene to prevent or delay the progression to dementia [16].

The results of this systematic review highlight the important protective role of cognitive reserve for cognitive impairment in healthy aging and the development of MCI. To take advantage of this potential protective factor, it is essential to establish universal and standardized diagnostic criteria for MCI and a standardized cognitive reserve assessment protocol, reflecting the complexity of this construct and considering its different indices in an aggregate manner, in order to obtain a more precise and complete assessment. The presence of a standardized and homogeneous assessment system for the diagnosis of MCI and the use of standardized instruments that comprehensively and aggregately evaluate the different indices of cognitive reserve would allow for greater comparability and generalizability of results. Cognitive and psychological treatments aiming to enhance cognitive reserve in patients with MCI could slow or halt the progression to dementia [53]. At the same time, interventions directed at increasing cognitive reserve in elderly populations at risk of cognitive impairment may be useful from a prevention perspective of future clinical pictures of MCI and dementia [54,55].

## Figures and Tables

**Table 1:** Databases and Script.

Script	Database	N°
(“cognitive reserve”) AND (MCI OR “mild cognitive impairment”) AND (elder* OR aged OR old* OR geriatric OR senior OR aging)	PSYCINFO	174
	MEDLINE	375
	SCOPUS	5111
	WEB OF SCIENCE	262

**Table 2:** Risk of Bias.

Study	Attrition Bias	Reporting Bias	Sample Bias	Measurement Bias
Algarabel et al. [30]	−	−	+	−
Andrejeva et al. [31]	−	−	−	−
Franzmeier et al. [32]	−	−	−	−
Geda et al. [20]	−	−	−	+
Kim et al. [26]	−	−	−	−
Liu et al. [25]	−	−	−	+
Malave [27]	−	−	−	−
Soldan et al. [28]	−	−	+	−
Solè-Padulles et al. [33]	−	−	+	−
Xu et al. [29]	−	−	+	−

“+” high risk of bias; “−” low risk of bias

**Table 3:** Diagnostic Criteria.

Authors	Diagnostic Criteria	Global Functioning	Subjective Cognitive Complaints	Cognitive Decline	Objective Cognitive Impairment	Normal Functional Abilities	Absence of Dementia	Normal Mental Status
Algarabel et al. [30]	Petersen [10]		✔		✔	✔	✔	✔
Andrejeva et al. [31]	Winblad et al. [11]; AACD [42]		✔	✔	✔	✔	✔	
Franzmeier et al. [32]	Petersen, [10]		✔		✔	✔	✔	✔
Geda et al. [20]	Mayo Clinic Revised [39]	CDR = 0 or 0.5	✔	✔		✔	✔	
Kim et al. [26]	Petersen et al. [35]		✔		✔	✔	✔	
Liu et al. [25]	DSM-IV [38]			✔	✔			
Malave [27]	Winblad et al. [11]			✔	✔	✔	✔	
Soldan et al. [28]	Albert et al. [40]	CDR = 0.5	✔	✔	✔	✔	✔	
Solè-Padullès et al. [33]	Petersen et al. [36]; Lopez et al. [37]		✔		✔	✔	✔	✔
Xu et al. [29]	NINCDS-ARDA [41]			✔	✔	✔	✔	

AACD = Aging-Associated Cognitive Decline; CDR = Clinical Dementia Rating Scale, DSM-IV = Diagnostical and Statical Manual of Mental Disorders IV; NINCDS-ADRDA = National Institute of Neurological and Communicative Disorders and Stroke-Alzheimer’s Disease and Related Disorders Association.

**Table 4:** Diagnostic Criteria.

Diagnostic Criteria		Authors
Albert et al. [40]	(1) Change in cognition. (2) Impairment in one or more cognitive domains. (3) Preservation of independence in functional abilities. (4) Not demented.	Soldan et al. [28]
Aging-Associated Cognitive Decline (AACD) [42]	Subjective impairment as reported by the patient or a reliable informant and considered a decline in a broad spectrum of cognitive domains. Deficits in relevant cognitive domains were indicated by neuropsychological test performance of at least 1 standard deviation below normal age and educational levels.	Andrejeva et al. [31]
Diagnostical and Statical Manual of Mental Disorders IV (DSM-IV) [38]	Cognitive decline - Self and/or informant report and impairment in objective cognitive tasks and/or - Evidence of decline over time on objective tasks. Preserved basic activities of daily living/minimal impairment in complex instrumental functions.	Liu et al. [25]
Lopez et al. [37]	(1) Memory decline according to clinical judgment, preferably corroborated by an informant. (2) Impaired memory function for age and education. (3) Preserved general cognitive function. (4) Intact activities of daily living. (5) Non-demented. (6) The memory impairment had to be of the episodic memory type defined by 1.5 S.D. below the control group mean, taking into account age and educational level. (7) Absence of psychiatric or medical causes accounting for these memory problems.	Solè-Padullès et al. [33]
Mayo Clinic Revised [39]	(1) Cognitive concern expressed by a physician, informant, participant, or nurse. (2) Cognitive impairment in one or more domains. (3) Normal functional activities. (4) No dementia.	Geda et al. [20]
National Institute of Neurological and Communicative Disorders and Stroke-Alzheimer’s Disease and Related Disorders Association (NINCDS-ARDA) [41]	(1) Dementia established by clinical examination and documented by the Mini-Mental Test, Blessed Dementia Scale, or some similar examination, and confirmed by neuropsychological tests. (2) Deficits in ≥2 areas of cognition. (3) Progressive worsening of memory and other cognitive functions. (4) No disturbance of consciousness. (5) Onset at age >40 to <90 years. (6) Absence of systemic disorders or other brain diseases that in and of themselves could account for the progressive deficits in memory and cognition.	Xu et al. [29]
Petersen [10]	(1) Memory complaint, preferably corroborated by an informant. (2) Objective memory impairment for age. (3) Relatively preserved general cognition for age. (4) Essentially intact activities of daily living. (5) Not demented.	Algarabel et al. [30]; Franzmeier et al. [32]
Petersen et al. [35]	(1) Memory complaint. (2) Normal activities of daily living. (3) Impairment in one or more tests in the Consortium to Establish a Registry for Alzheimer’s Disease (CERAD) neuropsychological assessment battery, as evidenced by scores of ≤−1.5 SD of age- and education-appropriate norms. (4) No dementia.	Kim et al. [26]
Petersen et al. [36]	(1) Memory decline according to clinical judgment, preferably corroborated by an informant. (2) Impaired memory function for age and education. (3) Preserved general cognitive function. (4) Intact activities of daily living. (5) Non-demented.	Solè-Padullès et al. [33]
Winblad et al. [11]	(1) The person is neither normal nor demented. (2) There is evidence of cognitive deterioration shown by either objectively measured decline over time and/or subjective report of decline by self and/or informant in conjunction with objective cognitive deficits. (3) Activities of daily living are preserved, and complex instrumental functions are either intact or minimally impaired.	Andrejeva et al. [31]; Malave, [27]

**Table 5:** Cognitive Functioning Assessment.

Authors	Global Cognitive Functioning	Visuospatial Abilities	Attention	Executive Functions	Language	Memory (Short Term)	Memory (Long Term)
Algarabel et al. [30]	- MMSE - GDS	- Praxis Imitation (TBR) - Praxis-Symbolic (TBR) - ROCF			- Vocabulary (WAIS) - Category Recall (TBR) - Verbal Fluency Task (TBR)	- Logical Memory (WMS) - Digit Span (WMS) - TAVEC	- Logical Memory (WMS) - ROCF - TAVEC
Andrejeva et al. [31]	- CERAD-NP						
Franzmeier et al. [32]	- MMSE - CDR						- ADNI-MEM
Geda et al. [20]	- CDR	- Block Design (WAIS-R) - Picture Completion (WAIS-R)		- TMT B (WAIS-R) - DSS (WAIS-R)	- BNT - Category Fluency		- Logical Memory (WMS) - Visual Reproduction (WMS) - AVLT
Liu et al. [25]	- MMSE - MoCA - GDS						
Kim et al. [26]	- CERAD-NP						
Malave [27]	- CDR	- Block Design - ROCF	- TMT A - Digit Span Forward	- TMT B - Stroop Task	- BNT - Animal Fluency Test		- CVLT - ROCF
Soldan et al. [28]				- DSS (WAIS-R)	- BNT	- Paired Associates Immediate Recall (WMS)	- Logical Memory (WMS)
Solè-Padullès et al. [33]							- Delayed Text Memory Test - Memory Recognition Task
Xu et al. [29]	- CERAD-NP						

ADNI-MEM = Alzheimer’s Disease Neuroimaging Initiative-Memory, BNT = Boston Naming Test, AVLT = Auditory Verbal Learning Test, CERAD-NP = Consortium to Establish a Registry for Alzheimer’s Disease-Neuropsychological test battery, CDR = Clinical Dementia Rating Scale, CVLT = California Verbal Learning Test, DSS = Digit Symbol Substitution, GDS = Global Deterioration Scale, MMSE = Mini Mental State Examination, MoCA = Montreal Cognitive Assessment, ROCF = Rey-Osterrieth Complex Figure, TAVEC = Test deprendizaje Verbal España-Complutense, TBR = Test Barcelona revisado, TMT A = Trail Making Test A, TMT B = Trail Making Test B, WAIS-R = Wechsler Adult Intelligence Scale-Revised, WMS = Wechsler Memory Scale.

**Table 6:** Results of Selected Studies.

Authors	Experimental Design	Nationality	Groups	N°	Sex (% F)	Age (SD)	Diagnostic Criteria	Cognitive Reserve Assessment	Cognitive Domain	Results
Algarabel et al. [30]	Cross-Sectional	Spain	MCI HC	37 39	67% 74%	74.95 69.75	Petersen [10]	Schooling, Job position, Leisure activities, Vocabulary (WAIS-III)	Global Cognitive Functioning, Visuospatial Abilities, Language, Memory	The MCI group exhibits worse performance in both memory tasks than HC. MCI with high CR exhibit better performance in implicit associative memory tasks than MCI with low CR.
Andrejeva et al. [31]	Cross-Sectional	Germany	aMCI aMCI+ HC	49 222 65	40% 51% 61%	66.57 (8.02) 72.27 (7.90) 69.56 (8.62)	Winblad et al. [11]; Levy [42]	Schooling	Global Cognitive Functioning	aMCI have a higher level of schooling than the aMCI+ group, which correlates with better cognitive functioning.
Franzmeier et al. [32]	Cross-Sectional	Germany	aMCI HC	76 36	47% 58%	71 (7.50) 75 (6.30)	Petersen [10]	Schooling, Verbal IQ (ANART)	Global Cognitive Functioning, Memory	aMCI with high CR have better performance in episodic memory tasks than MCI with low CR.
Geda et al. [20]	Cross-Sectional	USA	MCI HC	197 1124	41.1% 49.8%	83 80	Mayo Clinic Revised [39]	Cognitive stimulating activities	Global Cognitive Functioning, Visuospatial Abilities, Executive Functions, Language, Memory	Involvement in cognitively stimulating activities is associated with better cognitive functioning and a lower risk of MCI.
Liu et al. [25]	Longitudinal	China	MCI HC	21 18	52% 33%	68.5 64	DSM-IV [38]	Schooling, Job position	Global Cognitive Functioning	High CR is associated with lower MCI risk and better cognitive functioning.
Kim et al. [26]	Longitudinal	South Korea	MCI HC	22 22	77.3% 90%	74.23 (7.50) 71.45 (3.95)	Petersen et al. [35]	CRIq	Global Cognitive Functioning	MCI and HC differ in the “CRI-leisure time” section correlated with cognitive functioning.
Malave [27]	Longitudinal	USA	MCI HC	1058 1226	43.29% 47.39%	78 77	Winblad et al. [11]	LAQ, IQ (ANART, RPM)	Global Cognitive Functioning, Visuospatial Abilities, Attention, Executive Functions, Language, Memory	IQ and participation in cognitively stimulating activities (crossword puzzles and newspaper reading) are associated with a lower risk of MCI.
Soldan et al. [28]	Longitudinal	USA	MCI HC	66 237	51.5% 62%	63 (10.8) 55.7 (9.40)	Albert et al. [40]	Schooling, NART, Vocabulary (WAIS-III)	Executive Functions, Language, Memory	High CR is associated with a lower risk of MCI and better cognitive functioning. Among participants who develop MCI, more rapid cognitive decline is observed in those with higher CR.
Solé-Padullés et al. [33]	Cross-Sectional	Spain	aMCI HC	12 16	80% 45%	74.25 (6.18) 73.31 (4.90)	Petersen et al. [36]; Lopez et al. [37]	Schooling, Job position, Leisure activities, IQ	Memory	aMCI have lower CR and worse mnesic performance than HC.
Xu et al. [29]	Longitudinal	China	MCI HC	420 1182	72% 77%	82.2 78.8	NINCDS-ADRDA [41]	Schooling, Cognitive stimulating activities, Social stimulating activities	Global Cognitive Functioning	High CR is associated with a lower risk of MCI.

aMCI = amnesic Mild Cognitive Impairment, aMCI+ = amnesic Mild Cognitive Impairment multiple domains, ANART = American National Adult Reading Test, CR = Cognitive Reserve, CRI = Cognitive Reserve Index, CRIq = Cognitive Reserve Index questionnaire, CDR = Clinical Dementia Rating Scale, DSM-IV = Diagnostic and Statistical Manual of Mental Disorders IV, GDS = Global Deterioration Scale, HC = Healthy Controls, IQ = Intelligence Quotient, LAQ = Lifestyle Activity Questionnaire, MCI = Mild Cognitive Impairment, MMSE = Mini-Mental State Examination, MoCA = Montreal Cognitive Assessment, NART = National Adult Reading Test, NIA-AA = National Institute on Aging- Alzheimer’s Association, NINCDS-ADRDA = National Institute of Neurological and Communicative Disorders and Stroke-Alzheimer’s Disease and Related Disorders Association, RPM = Raven Progressive Matrices, SD = Standard Deviation, WAIS-III = Wechsler Adult Intelligence Scale-III, WAIS-R = Wechsler Adult Intelligence Scale-Revised.

## Data Availability

Not applicable.
